# Inhibitory Activity of Bioactive Phloroglucinols from the Rhizomes of *Dryopteris crassirhizoma* on *Escherichia coli* *β*-Glucuronidase: Kinetic Analysis and Molecular Docking Studies

**DOI:** 10.3390/metabo12100938

**Published:** 2022-10-02

**Authors:** Nguyen Viet Phong, Yan Zhao, Byung Sun Min, Seo Young Yang, Jeong Ah Kim

**Affiliations:** 1 Vessel-Organ Interaction Research Center, VOICE (MRC), College of Pharmacy, Kyungpook National University, Daegu 41566, Korea; 2 BK21 FOUR Community-Based Intelligent Novel Drug Discovery Education Unit, College of Pharmacy and Research Institute of Pharmaceutical Sciences, Kyungpook National University, Daegu 41566, Korea; 3 School of Pharmacy, Yantai University, Yantai 264005, China; 4 Drug Research and Development Center, College of Pharmacy, Daegu Catholic University, Gyeongsan 38430, Korea; 5 Department of Pharmaceutical Engineering, Sangji University, Wonju 26339, Korea

**Keywords:** *Dryopteris crassirhizoma*, phloroglucinols, *β*-glucuronidase, kinetic analysis, competitive inhibitor, molecular docking

## Abstract

Phloroglucinols—one of the major secondary metabolites in *Dryopteris crassirhizoma*—exhibit various pharmacological effects, such as antiviral, antioxidant, and antidiabetic activities. This study evaluated 30 phloroglucinols isolated from the rhizomes of *D. crassirhizoma* for their inhibitory activity on *β*-glucuronidase via in vitro assays. Among them, dimeric phloroglucinols **13**–**15** moderately inhibited *β*-glucuronidase, and trimeric phloroglucinols **26**–**28** showed strong inhibitory effects, with IC_50_ values ranging from 5.6 to 8.0 μM. Enzyme kinetic analysis confirmed all six active compounds to be in a competitive mode of inhibition. Molecular docking simulations revealed the key binding interactions with the active site of *β*-glucuronidase protein and the binding mechanisms of these active metabolites. Our results suggest that the rhizomes of *D. crassirhizoma* and trimeric compounds **26**–**28** may serve as potential candidates for discovering and developing new *β*-glucuronidase inhibitors.

## 1. Introduction

*β*-Glucuronidases hydrolyze *β*-glucuronic acid (GlcA)-containing carbohydrates to release GlcA and play a role in endosterol and bilirubin metabolism [1]. The Carbohydrate-Active Enzymes (CAZy) database classifies *β*-glucuronidases into three glycoside hydrolase (GH) families, i.e., GH1, GH2, and GH79, according to their amino acid sequences [2]. GH2, a member of the GH-A clan, contains *exo*-acting enzymes such as *β*-galactosidases, *β*-mannosidases, and *β*-glucuronidases [3]. *β*-Glucuronidases are frequently found in microbes, plants, and various mammalian tissues, but are most prevalent in the digestive system [3]. The first 3D crystal structure of human GH2 *β*-glucuronidase was identified by Jain et al., in 1996 [4], followed by a report on the structure of *Escherichia coli* GH2 *β*-glucuronidase by Wallace et al., in 2010 [5]. *β*-Glucuronidase is essential for the enterohepatic circulation of certain medications and endogenous metabolites. The glucuronides of several clinical drugs, such as indomethacin and irinotecan, may be degraded by *β*-glucuronidase from bacteria in the intestinal tract to produce aglycones with considerable toxicity and cause significant gastrointestinal side effects [6]. As a result, the discovery of *β*-glucuronidase inhibitors has now focused on treating irinotecan-induced gastrointestinal diarrhea. In addition, a previous report revealed that the occurrence and development of tumor cells, including survival, proliferation, invasion, and metastasis, are closely correlated with *β*-glucuronidase activity [7]. The expression of *β*-glucuronidase is higher in tumor cells than in normal cells, including breast, ovary, lung, and colon tumor cells [8]. Thus, *β*-glucuronidase has also been considered a potential biomarker for the clinical diagnosis and treatment of cancers.

From ancient times to the present day, natural products have played a key role in the discovery of new drugs, and the research, investigation, and development of active metabolites from natural herbal products are and will continue to be important sources of potential therapeutic agents for use against various diseases [9,10]. *Dryopteris crassirhizoma*, a member of the Dryopteridaceae family, is a pteridophyte. Its rhizomes are one the most widely used traditional herbal medicines in Northeast Asia and China. They have been used to treat parasite infestation, hemorrhage, and common cold [11]. *D*. *crassirhizoma* was originally employed as an antihelminthic herbal treatment against *Diphyllobothrium latum* and as an anti-infection agent to cure common cold and flu [12,13]. *D*. *crassirhizoma* has recently been reported to exhibit antiviral, antiplatelet, antioxidant, antibacterial, and antitumor activities [14,15,16,17,18]. The major secondary metabolites of *D*. *crassirhizoma* are phloroglucinols and their derivatives, which demonstrate various pharmacological effects. Approximately 50 phloroglucinols have been isolated and structurally elucidated from *D*. *crassirhizoma*, and can be divided into five groups based on the number of monomers in their chemical structures [19]. Among these, dimeric phloroglucinols flavaspidic acid AP and its derivatives flavaspidic acid AB, flavaspidic acid PB, and flavaspidic acid BB have been reported to exhibit significant xanthine oxidase inhibitory activities with IC_50_ values of 6.3–20.9 μM [20]. Norflavaspidic acid PB and flavaspidic acid PB have also been reported to inhibit melanin production in B16F10 murine melanoma cells with IC_50_ values of 35.7 and 38.9 μM, respectively [21]. In recent years, trimeric phloroglucinols have attracted considerable attention from both chemists and pharmacists. The trimeric compounds filixic acid ABA and nortrisflavaspidic acid ABB exhibited a strong inhibitory effect on neuraminidase of influenza virus H5N1, with IC_50_ values of 29.6 and 51.5 μM, respectively [22]. Furthermore, nortrisflavaspidic acid ABB, trisflavaspidic acid ABB, and trisflavaspidic acid BBB are considered promising treatments for type 2 diabetes due to their significant activity toward the PTP1B enzyme [23]. Although many biological activities of secondary metabolites from the rhizomes of *D*. *crassirhizoma* have been studied, the inhibitory activity of phloroglucinols on *β*-glucuronidase has not been reported.

**Figure 1:**
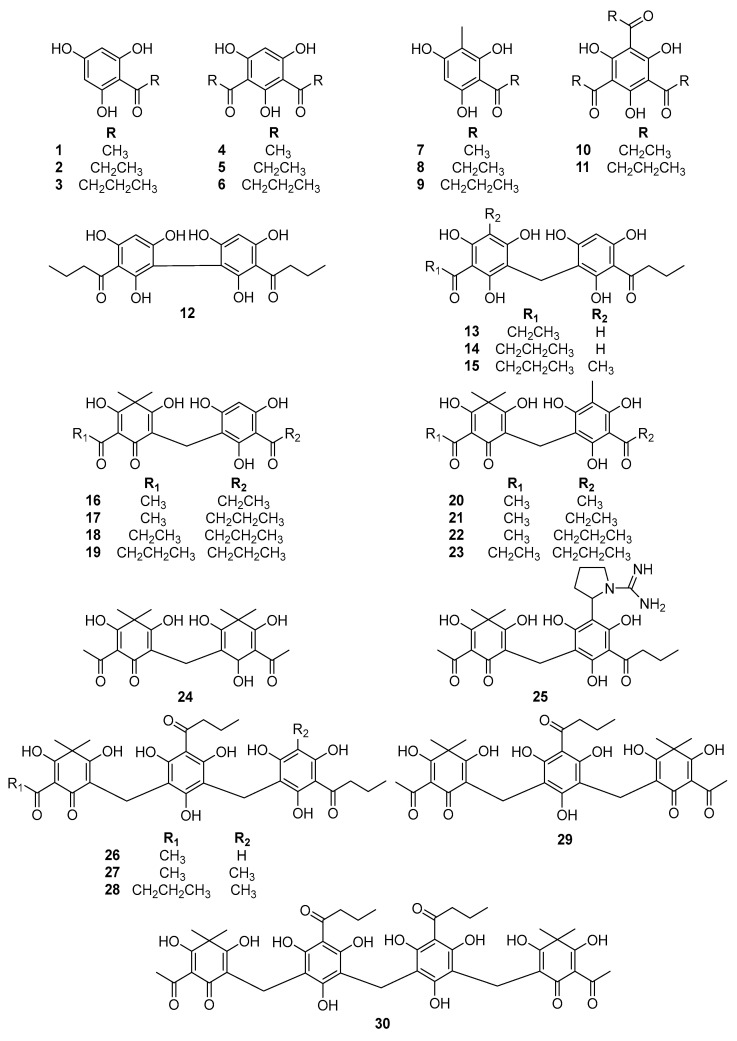
Chemical structures of phloroglucinols from *D. crassirhizoma* (**1**–**30**).

In our previous study, we reported the isolation of 30 phloroglucinols from a methanol extract of *D*. *crassirhizoma* rhizomes [23]. Based on analysis of various modern spectroscopic data, such as high-resolution mass spectrometry, 1D and 2D nuclear magnetic resonance (NMR), spectroscopy as well as comparison with corresponding results in the literature, the chemical structures of the isolated compounds were identified as 2,4,6-trihydroxy-acetophenone (**1**), 1-(2,4,6-trihydroxy-phenyl)-propan-1-one (**2**), phlorobutyrophenone (**3**), 5-acetyl-2,4,6-trihydroxyacetophenone (**4**), 1,3-(2,4,6-trihydroxyphenyl)dipropanone (**5**), 1,3-(2,4,6-trihydroxyphenyl)dibutanone (**6**), 1-(2,4,6-trihydroxy-3-methylphenyl)propanone (**7**), 1-(2, 4, 6-trihydroxy-3-methylphenyl) butanone (**8**), 1-(2, 4, 6-trihydroxy-3-methylphenyl) pentanone (**9**), tripropionylphloroglucinol (**10**), tributyrylphloroglucinol (**11**), bis-phlorobutyrophenone (**12**), dryopidin PB (**13**), methylene-bis-phlorobutyrophenone (**14**), araspidin BB (**15**), norflavaspidic acid AP (**16**), norflavaspidic acid AB (**17**), norflavaspidic acid PB (**18**), norflavaspidic acid BB (**19**), flavaspidic acid AA (**20**), flavaspidic acid AP (**21**), flavaspidic acid AB (**22**), flavaspidic acid PB (**23**), albaspidin AA (**24**), dryopcrassirine AB (**25**), nortrisflavaspidic acid ABB (**26**), trisflavaspidic acid ABB (**27**), trisflavaspidic acid BBB (**28**), filixic acid ABA (**29**), and dryocrassin ABBA (**30**) (Figure 1). All isolated compounds were evaluated for their PTP1B inhibitory activities through in vitro assays, and some exhibited significant inhibitory effects [23].

As part of our ongoing study to investigate the biological activity of secondary metabolites from *D*. *crassirhizoma*, in this study, all the phloroglucinols isolated (**1**–**30**) were evaluated for their in vitro *β*-glucuronidase inhibitory effect. Kinetic analysis and molecular docking studies were also performed to understand the inhibition type, binding efficiency, and binding interaction of the active compounds with *β*-glucuronidase.

## 2. Results

### 2.1. Inhibitory Effect of Phloroglucinols on β-Glucuronidase

**Figure 2:**
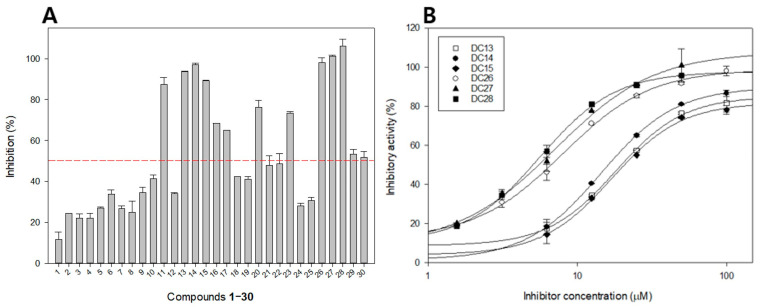
Inhibitory activity of compounds **1**–**30** at 100 µM (**A**) and IC_50_ values of active phloroglucinols **13**–**15** and **26**–**28** on *β*-glucuronidase (**B**).

The inhibitory effect of all isolated phloroglucinols (**1**–**30**) against *β*-glucuronidase was evaluated using 4-nitrophenyl *β*-d-glucuronide (PNPG) as a substrate. The results are expressed as IC_50_ values, depicted in Figure 2 and Table 1. The results revealed that no inhibitory activity of *β*-glucuronidase (IC_50_ >100 µM) for monomeric phloroglucinols (**1**–**10**), except for tributyrylphloroglucinol (**11**) with three butyryl groups in the side chain, which showed moderate *β*-glucuronidase inhibitory effect with an IC_50_ value of 43.0 ± 2.9 µM. These results suggest that the number of butyryl groups in the chemical structures of phloroglucinols may significantly influence the inhibitory effect of *β*-glucuronidase.

Dimeric phloroglucinols (**13**–**23**) exhibited more potent *β*-glucuronidase inhibition than monomeric phloroglucinols. Among them, compounds **13**, **14**, and **15** with at least one butyryl group linked to phenol rings exhibited a strong inhibitory effect on *β*-glucuronidase with IC_50_ values of 18.1 ± 1.5, 14.4 ± 0.6, and 17.0 ± 2.5 µM, respectively, which were higher than those of the positive control d-saccharic acid 1,4-lactone (DSA, IC_50_ = 23.4 ± 1.5 µM). The other dimeric compounds showed weak or no *β*-glucuronidase inhibitory activity (IC_50_ values ranging from 64.4 ± 3.5 to >100 µM).

Among all the isolated compounds, trimeric phloroglucinols (**26**–**28**) displayed the strongest inhibitory effect against *β*-glucuronidase. In vitro assessments revealed that nortrisflavaspidic acid ABB (**26**), trisflavaspidic acid ABB (**27**), and trisflavaspidic acid BBB (**28**), with two butyryl side chains linked to phenol rings in phloroglucinol trimeric structures, significantly exhibited PTP1B [23]. In this study, these molecules also showed significant *β*-glucuronidase inhibitory activity with IC_50_ values of 8.0 ± 1.8, 7.1 ± 2.6, and 5.6 ± 1.1 µM, respectively, which was more potent than that of the positive control DSA. Compounds **26**–**28** with two phloroglucinol units linked with a 2,5-cyclohexadienone ring exhibited a much stronger *β*-glucuronidase inhibitory effect than trimeric compound **29** with only one phloroglucinol unit linked with two 2,5-cyclohexadienone rings (IC_50_ = 90.5 ± 2.7 µM), suggesting that the number of phloroglucinol units in the chemical structure of phloroglucinols may positively affect its *β*-glucuronidase inhibition. Interestingly, this suggestion is fully consistent with what was observed in dimeric group. The *β*-glucuronidase inhibitory effect of tetrameric phloroglucinol dryocrassin ABBA (**30**) (IC_50_ = 94.9 ± 2.5 µM) was decreased compared to that of the trimeric group. Structure–activity relationship analysis suggested that the trimeric skeleton of phloroglucinol plays a vital role in its *β*-glucuronidase inhibitory effect.

### 2.2. Inhibition Kinetics of Active Compounds on β-Glucuronidase

**Figure 3:**
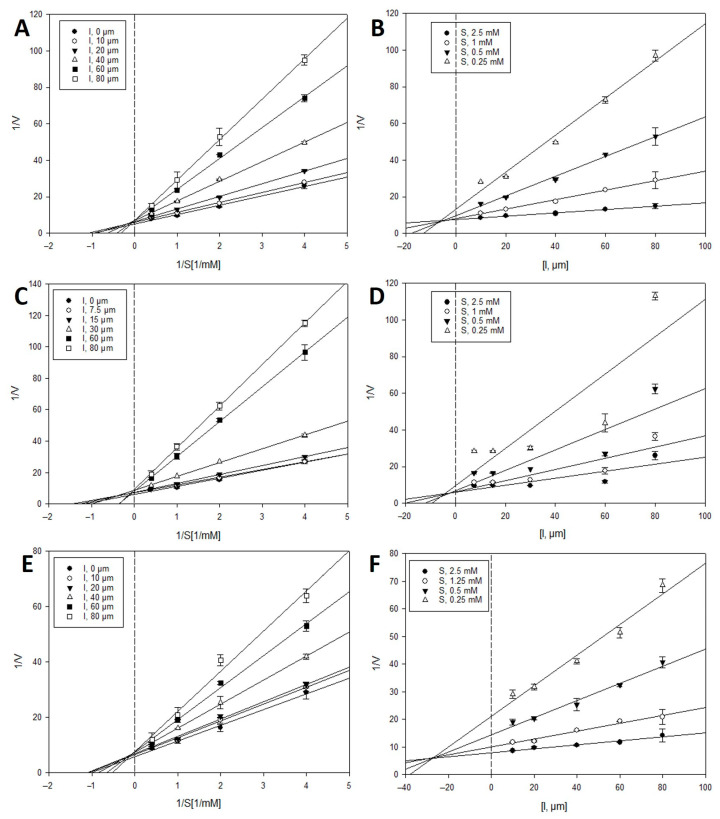
Lineweaver–Burk plots (**A**,**C**,**E**) and Dixon plots (**B**,**D**,**F**) for the *β*-glucuronidase inhibition of dimeric phloroglucinols **13**–**15**, respectively.

**Figure 4:**
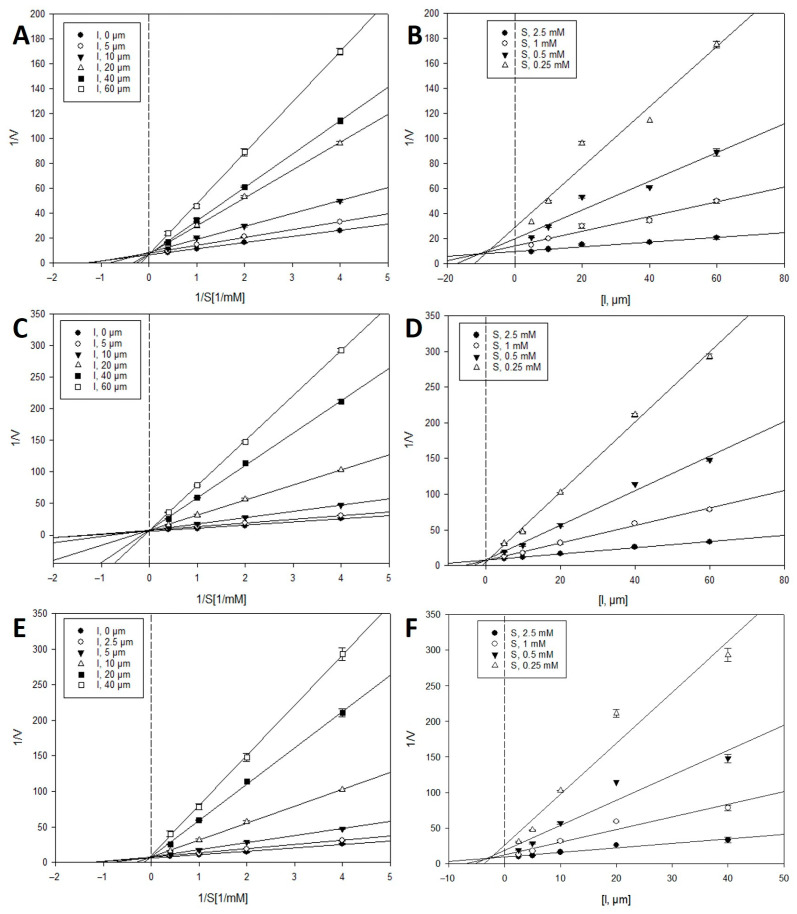
Lineweaver–Burk plots (**A**,**C**,**E**) and Dixon plots (**B**,**D**,**F**) for the *β*-glucuronidase inhibition of trimeric phloroglucinols **26**–**28**, respectively.

To investigate the types of enzyme inhibition and inhibition constants (*K_i_*), kinetic analyses were performed at various concentrations of the PNPG substrate and inhibitors (**13**–**15** and **26**–**28**) using the Lineweaver–Burk and Dixon analysis methods [24,25]. In the Lineweaver–Burk method, the intersection of the family of straight lines in the xy region indicated mixed inhibition. In contrast, uncompetitive or competitive inhibition was demonstrated by the lines crossing at the same point on the *x*- or *y*-axes, respectively. As shown in Figure 3 and Figure 4, and Table 2, the Lineweaver–Burk plots showed a family of straight lines that crossed each other on the *y*-axis or 1/V axis. These results indicate that the inhibitory behavior of active compounds **13**–**15** and **26**–**28** toward *β*-glucuronidase was a competitive type of inhibition. According to the Dixon plots, the *K_i_* values of compounds **13**–**15** and **26**–**28** were 6.3, 4.3, 25.8, 7.5, 0.5, and 2.8 μM, respectively.

### 2.3. Molecular Docking Simulation of β-Glucuronidase Inhibition

**Figure 5:**
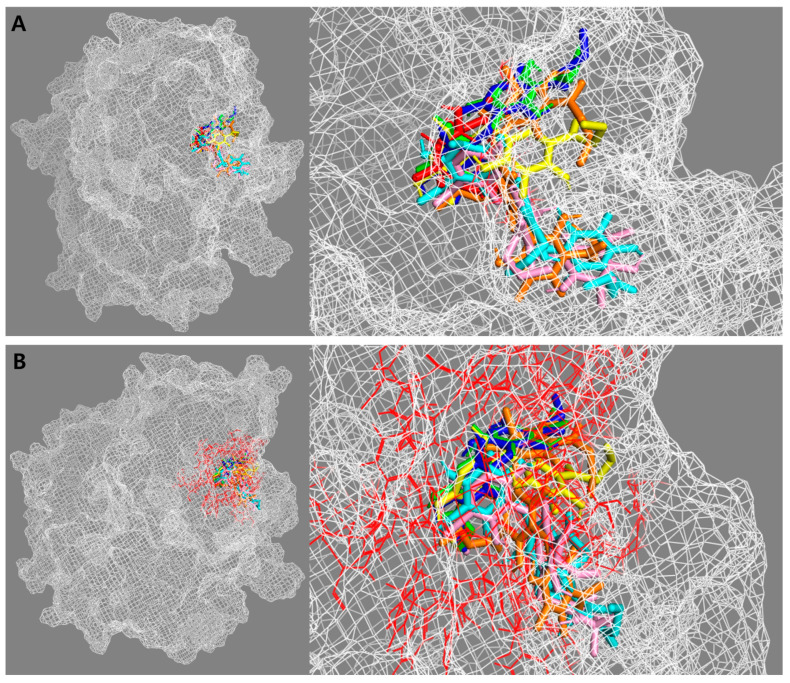
The docking results of the substrate and active compounds **13**–**15** and **26**–**28** ((**A**) substrate: red, compounds **13**: green, **14**: blue, **15**: yellow, **26**: pink, **27**: cyan, and **28**: orange) and the active site of the *β*-glucuronidase enzyme ((**B**) red site).

To understand the binding efficiency and interaction of active compounds **13**–**15** and **26**–**28** with the *β*-glucuronidase enzyme, a molecular docking simulation was performed using AutoDock 4.2 (Scripps Research Institute, La Jolla, CA, USA). The results were visualized using BIOVIA Discovery Studio 21.1 (BIOVIA, Dassault Systèmes, San Diego, CA, USA) and PyMOL 2.5 (Schrödinger, Inc., New York, NY, USA). The kinetic results suggest that all test compounds displayed a competitive type of inhibition. Therefore, compounds **13**–**15** and **26**–**28** could bind to the active site of *β*-glucuronidase, where the substrate molecule binds and carries a chemical reaction. Thus, PNPG was docked as a native ligand into the *β*-glucuronidase enzyme (PDB ID: 6LEL) to validate and optimize the docking operation. The docking results of the substrate and active compounds, as well as the active site of the *β*-glucuronidase enzyme, are presented in Figure 5.

**Figure 6:**
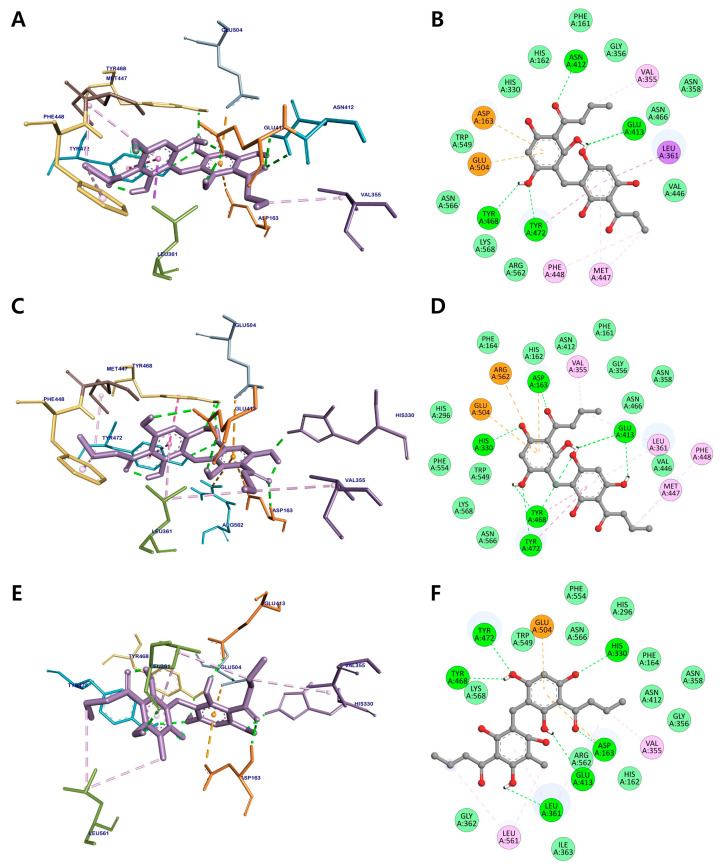
Three-dimensional molecular docking (**A**,**C**,**E**) and 2D interaction diagrams (**B**,**D**,**F**) of *β*-glucuronidase inhibition by dimeric phloroglucinols **13**–**15**, respectively. Green: hydrogen bonding interactions, light green: van der Waals interactions, pink: hydrophobic interactions, and orange: electrostatic interactions with the corresponding amino acid residues of *β*-glucuronidase.

According to our docking results, dimeric compounds **13**–**15** could bind to the active site of *β*-glucuronidase, with binding energy values of −8.22, −8.49, and −8.02 kcal/mol, respectively (Figure 6 and Table 3). The hydroxy groups of the three active compounds shared Glu413, Tyr468, and Tyr472 residues via hydrogen-bonding interactions, while their aromatic rings formed a π-anion interaction with Glu504. The butyryl groups of compounds **13** and **14** established alkyl and π-alkyl interactions with Val355, Met447, and Phe448 in the active site of *β*-glucuronidase, respectively. The carbonyl and hydroxy groups of **15** also displayed hydrogen bonding interactions with Asp163, His330, and Leu361. The other residues, including Gly356, Asn358, Val446, Asn466, Trp549, Arg562, Asn566, and Lys568, from different sites of *β*-glucuronidase, were bound to three active dimeric phloroglucinols through van der Waals interactions.

**Figure 7:**
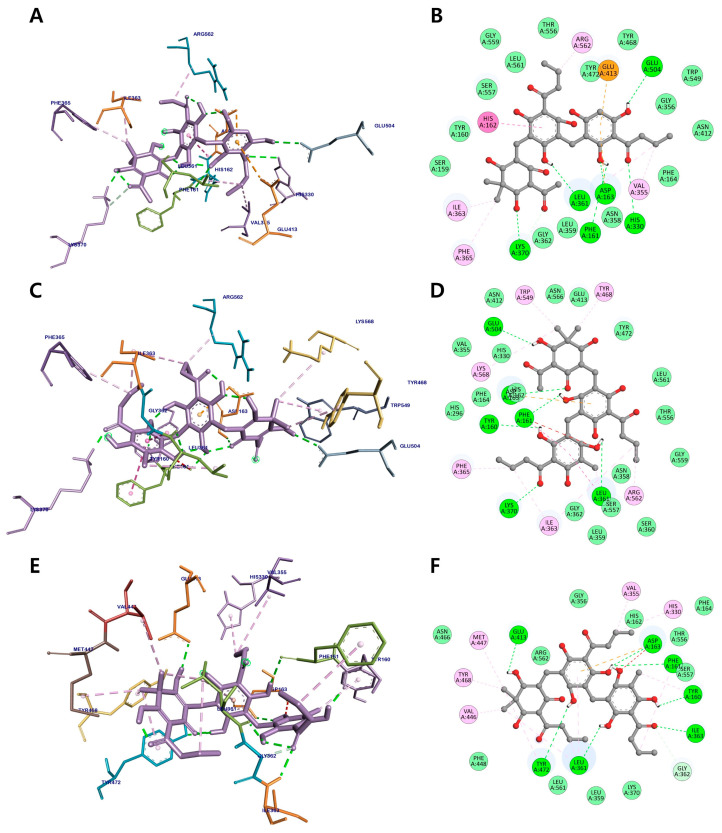
Three-dimensional molecular docking (**A**,**C**,**E**) and 2D interaction diagrams (**B**,**D**,**F**) of *β*-glucuronidase inhibition by trimeric phloroglucinols **26**–**28**, respectively.

Figure 7 and Table 3 display the binding mechanism of trimeric phloroglucinols **26**–**28** and the active site of *β*-glucuronidase with binding energies of −10.67, −10.26, and −9.56 kcal/mol, respectively. The lower binding energies found in these three active metabolites indicate that trimeric phloroglucinols have higher inhibitory activity than the dimeric group. The hydroxy groups of the three trimeric compounds share the residues Phe161, Asp163, His330, Leu361, Gly362, Lys370, and Glu504 via hydrogen-bond interactions. The methyl and butyryl groups of **26** displayed alkyl and π-alkyl interactions with Ile363, Phe365, Val355, and Arg562, whereas the aromatic rings formed amide-π and π-anion interactions with His162 and Glu413, respectively. The methyl and butyryl groups of **27** establish alkyl interactions with Ile363, Arg562, and Lys568 and π-alkyl interactions with Phe365, Tyr468, and Trp549. In addition, residues His330, Val355, Val446, Met447, and Tyr468 were bound to the methyl and butyryl groups of **28** via alkyl and π-alkyl interactions. These results, together with those of the *β*-glucuronidase inhibition assay, indicate the importance of butyryl groups in the chemical structure of phloroglucinols for the inhibition of *β*-glucuronidase.

## 3. Discussion

*β*-Glucuronidase, one of the enzymes involved in the hydrolysis of conjugated compounds, has received significant attention in recent years because of its role in removing hazardous chemicals from the body in the form of glucuronides [26]. The asymmetric unit of the *E*. *coli β*-glucuronidase structure (~139 kDa) consists of two monomers with 597 amino acid residues and is divided into three main regions: the N-terminal (180 residues) resembles the sugar-binding domain of the GH2 family, the C-terminal domain (residues 274 to 603) forms an αβ barrel and has two catalytic residues in the active site—Glu413 (catalytic acid) and Glu504 (catalytic nucleophile)—and the region between the N- and C-terminal domains has an immunoglobulin-like *β*-sandwich domain similar to other members of the GH2 family [5]. Especially, the bacterial loop (residues 360–376) found in *E*. *coli β*-glucuronidase, which is missing from the human orthologue, was reported to be a key factor for the complete activity of *β*-glucuronidase and its selective inhibition [5,27].

Virtual screening based on molecular docking has been the most popular method for structure-based drug discovery since 1982 [28]. Researchers have characterized small-molecule activity at target protein-binding sites and disclosed basic biochemical processes using molecular docking models to depict the atomic-level interaction between a small molecule and a protein [29]. The two main processes in the docking procedure are predicting the ligand shape, location, and orientation at these sites and determining the binding energy [30]. The benefits of virtual screening include small search space, low cost, and high flexibility. These factors can aid in the rapid discovery of a possible target protein inhibitor. Herein, we describe phloroglucinols isolated from the rhizomes of *D*. *crassirhizoma* that are potential natural therapeutic agents for inhibiting *β*-glucuronidase, determined via a combination of in vitro assays and virtual screening techniques.

All the phloroglucinols isolated (**1**–**30**) were evaluated for *β*-glucuronidase inhibitory activity. Of these, dimeric compounds **13**–**15** showed moderate effects on *β*-glucuronidase, with IC_50_ values ranging from 14.4 to 18.1 μM. Trimeric compounds **26**–**28** exhibited strong inhibitory effects on *β*-glucuronidase, with IC_50_ values ranging from 5.6 to 8.0 μM. In comparison, the monomeric group showed weak or no *β*-glucuronidase inhibition activity (IC_50_ values >100 μM). Examination of structure–activity relationships among the active compounds suggested that the presence of three aromatic rings in the chemical structure of phloroglucinols is important for potent *β*-glucuronidase inhibitory activity. Our previous report emphasized the importance of the trimeric chemical structure of phloroglucinols in positively increasing the metabolites efficiency. Kinetic analysis and molecular docking simulations of the active compounds were performed to investigate the binding position and cause of the activity difference. A comparison of the docking results revealed that trimeric phloroglucinols **26**–**28** exhibited lower binding energies than the dimeric phloroglucinols, as evidenced by the stronger *β*-glucuronidase inhibitory activity of the trimeric group in comparison to the dimeric group. Dimeric compounds **13**–**15** displayed linkages with two important catalytic residues, Glu413 and Glu504, in the active site through hydrogen bonding and π-anion interactions, respectively. Trimeric compounds **26**–**28** interacted with Glu413 and Glu504 and tightly interacted with Leu361, Gly362, Ile363, and Lys370 in the bacterial loop of *E*. *coli β*-glucuronidase via hydrogen bonds, van der Waals forces, and hydrophobic interactions, which might explain their significant activity against *β*-glucuronidase.

## 4. Materials and Methods

### 4.1. Chemicals and Reagents

β-Glucuronidase from *E. coli* (EC 3.2.1.31, G7396) and d-saccharic acid 1,4-lactone (DSA, S0375) were purchased from Sigma-Aldrich Co. (St. Louis, MO, USA). 4-Nitrophenyl *β*-d-glucuronide (PNPG, N0618) was purchased from Tokyo Chemical Industry Co., Ltd. (Tokyo, Japan).

### 4.2. Assay for β-Glucuronidase Inhibitory Activity

β-Glucuronidase inhibitory activity was assayed in accordance with a previously described protocol [31]. Briefly, the reaction was started by adding 130 μL *β*-glucuronidase (50 U/mL) to 100 mM phosphate buffer adjusted to pH 6.8. The solution was mixed in 96-well plates containing 20 μL of DMSO or the compound was dissolved in DMSO, followed by incubation at 37 °C for 20 min in 50 μL PNPG (1 mM for the inhibition assay). *β*-Glucuronidase inhibitory activity was determined by monitoring the amount of 4-nitrophenol released from the substrate PNGP at 405 nm using a UV-vis spectrophotometer (VersaMax, Molecular Devices, San Jose, CA, USA). DSA, an inhibitor of *β*-glucuronidase, was used as the positive control. Percentage inhibition (%) was calculated using the following equation:

inhibitory activity (%) = [(Δcontrol − Δsample)/Δcontrol] × 100,

where control and sample are the intensities of the control and inhibitor after 20 min, respectively.

IC_50_ values were calculated using SigmaPlot 10.0 (Systat Software Inc., Palo Alto, CA, USA).

### 4.3. Inhibition Kinetic Analysis with β-Glucuronidase

To identify the inhibition mode of the active compounds on *β*-glucuronidase, two enzyme kinetic methodologies, the Lineweaver-Burk and Dixon methods, were used [24,25]. The mode of *β*-glucuronidase inhibition was determined using a Lineweaver–Burk plot (or double reciprocal plot) at various concentrations of the substrate in the absence or presence of the test compound (concentrations: 0, 10, 20, 40, 60, and 80 µM for **13** and **15**; 0, 7.5, 15, 30, 60, and 80 µM for **14**; 0, 5, 10, 20, 40, and 60 µM for **26** and **27**; and 0, 2.5, 5, 10, 20, and 40 µM for **28**). The enzymatic reactions at various concentrations of the test compounds were evaluated by monitoring the effects of different substrate concentrations using Dixon plots (or single reciprocal plots) (0.25, 0.5, 1, and 2.5 mM). *Ki* values were determined by analyzing the Dixon plot, where the *x*-axis value was taken as *Ki*. The analysis and visualization of the experimental data were performed using SigmaPlot 10.0 (Systat Software Inc., Palo Alto, CA, USA).

### 4.4. Molecular Docking Simulation

Molecular docking simulations were performed to understand the binding efficiency and interaction of active compounds with *β*-glucuronidase using AutoDock 4.2 (Scripps Research Institute, La Jolla, CA, USA) [32], as per our previously described protocol [23,31]. The 3D X-ray crystallographic structure of *E*. *coli β*-glucuronidase (PDB ID: 6LEL) was obtained from the RCSB Protein Data Bank database at a resolution of 2.5 Å [33]. The 3D structures of the test compounds were built and minimized using Spartan’18 (Wavefunction Inc., Irvine, CA, USA). The pdb file of 6LEL protein was added to hydrogens and charged by computing Gasteiger charges using AutoDockTools 1.5.6 (Scripps Research Institute, La Jolla, CA, USA). The grid box was set (grid points X.Y.Z 60.60.60) to include the loop containing the active site. All test compounds were docked into the box with default values of Lamarckian genetic algorithm parameters, except for the number of genetic algorithms (runs = 50) and the maximum number of energy evaluations (25,000,000). The docking simulation results were visualized using Discovery Studio Visualizer 21.1 (BIOVIA, Dassault Systèmes, San Diego, CA, USA) and PyMOL 2.5 (Schrödinger, Inc., New York, NY, USA) for the 3D molecular docking model and 2D ligand interaction diagram.

### 4.5. Statistics

All results are presented as means ± standard error of the mean (SEM) of at least three independent experiments. Significance was analyzed using one-way ANOVA and Duncan’s test (Systat Software Inc., Palo Alto, CA, USA) and was noted at *p* < 0.05.

## 5. Conclusions

In conclusion, this is the first report of the inhibitory effect against *β*-glucuronidase of phloroglucinols isolated from the rhizomes of *D*. *crassirhizoma*. Our results showed that nortrisflavaspidic acid ABB (**26**), trisflavaspidic acid ABB (**27**), and trisflavaspidic acid BBB (**28**) exhibited strong inhibitory activity on *β*-glucuronidase with IC_50_ values of 8.0, 7.1, and 5.6 μM, respectively, whereas the dimeric compounds **13**–**15** showed a moderate *β*-glucuronidase inhibitory effect. Structure–activity relationship analysis confirmed the importance of the trimeric skeleton in the chemical structure of phloroglucinols for their biological activity. Kinetic studies indicated that all the active compounds displayed competitive inhibition of *β*-glucuronidase. Moreover, molecular docking simulations demonstrated that these active compounds could tightly bind to the active sites of *β*-glucuronidase with negative binding energies, in agreement with the results of kinetic analysis. Our in vitro and in silico results suggest that *D*. *crassirhizoma* rhizomes and their phloroglucinols have great potential as natural herbal *β*-glucuronidase inhibitors.

## Figures and Tables

**Table 1:** Inhibitory activity of all isolated phloroglucinols **1**–**30** on *β*-glucuronidase.

Compounds	Inhibition of Compounds on *β*-glucuronidase	
	Inhibition at 100 μM (%)	IC_50_ (μM) ^1^
**1**	11.6 ± 3.9	>100
**2**	24.4 ± 0.2	>100
**3**	22.0 ± 2.1	>100
**4**	22.1 ± 2.4	>100
**5**	27.1 ± 0.4	>100
**6**	33.7 ± 2.3	>100
**7**	26.7 ± 1.5	>100
**8**	24.9 ± 5.5	>100
**9**	34.7 ± 2.4	>100
**10**	41.4 ± 1.7	>100
**11**	87.3 ± 3.4	43.0 ± 2.9
**12**	34.1 ± 0.5	>100
**13**	93.7 ± 0.3	18.1 ± 1.5
**14**	97.1 ± 0.7	14.4 ± 0.6
**15**	89.1 ± 0.3	17.0 ± 2.5
**16**	68.3 ± 0.2	64.4 ± 3.5
**17**	65.1 ± 0.1	67.0 ± 1.3
**18**	42.2 ± 0.1	>100
**19**	41.0 ± 1.4	>100
**20**	76.1 ± 3.4	75.7 ± 1.2
**21**	47.9 ± 4.7	>100
**22**	48.7 ± 4.8	>100
**23**	73.5 ± 0.7	76.5 ± 1.5
**24**	28.1 ± 1.2	>100
**25**	30.7 ± 1.7	>100
**26**	98.09 ± 2.5	8.0 ± 1.8
**27**	101.2 ± 0.6	7.1 ± 2.6
**28**	106.2 ± 3.4	5.6 ± 1.1
**29**	53.4 ± 2.3	90.5 ± 2.7
**30**	51.8 ± 2.7	94.9 ± 2.5
DSA ^2^	79.0 ± 0.3	23.4 ± 1.5

^1^ The values (μM) indicate 50% inhibitory effects. All data are expressed as the mean ± SEM of three independent experiments. ^2^ Positive control.

**Table 2:** Kinetic parameters of active compounds against *β*-glucuronidase.

Compounds	Inhibition Type ^1^	*K_i_* (μM) ^2^
**13**	Competitive	6.3
**14**	Competitive	4.3
**15**	Competitive	25.8
**26**	Competitive	7.5
**27**	Competitive	0.5
**28**	Competitive	2.8

^1^ Determined by Lineweaver–Burk plots. ^2^ Determined by Dixon plots.

**Table 3:** Docking energies and binding site interactions of active phloroglucinols **13**–**15** and **26**–**28** with *β*-glucuronidase.

Compounds	Binding Energy (kcal/mol)	Hydrogen Bonds	van der Waals Interactions	Hydrophobic Interactions	Electrostatic Interactions
**13**	−8.22	Asn412, Glu413, Tyr468, Tyr472	Phe161, His162, His330, Gly356, Asn358, Val446, Asn466, Trp549, Arg562, Asn566, Lys568	Leu361 (π-σ) Val355 (alkyl) Met447 (π-alkyl) Phe448 (π-alkyl)	Asp163 (π-anion) Glu504 (π-anion)
**14**	−8.49	Asp163, His330, Glu413, Tyr468, Tyr472	Phe161, His162, Phe164, Gly356, Asn358, Asn412, Val446, Asn466, Trp549, Asn566, Lys568	Val355 (alkyl) Leu361 (alkyl) Met447 (π-alkyl) Phe448 (π-alkyl)	Glu504 (π-anion) Glu562 (π-cation)
**15**	−8.02	Asp163, His330, Leu361, Glu413, Tyr468, Tyr472	His162, Phe164, His296, Gly356, Asn358, Gly362, Ile363, Asn412, Trp549, Phe554, Arg562, Asn566, Lys568	Val355 (alkyl) Leu561 (alkyl)	Glu504 (π-anion)
**26**	−10.67	Phe161, Asp163, His330, Leu361, Lys370, Glu504	Ser159, Tyr160, Phe164, Gly356, Asn358, Leu359, Gly362, Asn412, Tyr468, Tyr472, Trp549, Thr556, Ser557, Gly559, Leu561	His162 (amide-π) Ile363 (alkyl) Val355 (alkyl) Arg562 (alkyl) Phe365 (π-alkyl)	Glu413 (π-anion)
**27**	−10.26	Tyr160, Phe161, Asp163, Leu361, Gly362, Lys370, Glu504	His162, Phe164, His296, His330, Val355, Asn358, Leu359, Ser360, Gly362, Asn412, Glu413, Tyr472, Thr556, Ser557, Gly559, Leu561, Asn566	Ile363 (alkyl) Arg562 (alkyl) Lys568 (alkyl) Phe365 (π-alkyl) Tyr468 (π-alkyl) Trp549 (π-alkyl)	
**28**	−9.56	Tyr160, Phe161, Asp163, Leu361, Gly362, Ile363, Glu413, Tyr472	His162, Phe164, Gly356, Leu359, Lys370, Phe448, Asn466, Thr556, Ser557, Leu561, Arg562	Val355 (alkyl) Val446 (alkyl) Met447 (alkyl) His330 (π-alkyl) Tyr468 (π-alkyl)	

## Data Availability

Not applicable.

## References

[1] Michikawa M., Ichinose H., Momma M., Biely P., Jongkees S., Yoshida M., Kotake T., Tsumuraya Y., Withers S.G., Fujimoto Z. (2012). Structural and biochemical characterization of glycoside hydrolase family 79 β-glucuronidase from *A**cidobacterium capsulatum*. J. Biol. Chem..

[2] Cantarel B.L., Coutinho P.M., Rancurel C., Bernard T., Lombard V., Henrissat B. (2009). The carbohydrate-active enzymes database (CAZy): An expert resource for glycogenomics. Nucleic Acids Res..

[3] Sun C.-P., Yan J.-K., Yi J., Zhang X.-Y., Yu Z.-L., Huo X.-K., Liang J.-H., Ning J., Feng L., Wang C. (2020). The study of inhibitory effect of natural flavonoids toward *β*-glucuronidase and interaction of flavonoids with *β*-glucuronidase. Int. J. Biol. Macromol..

[4] Jain S., Drendel W.B., Chen Z.-W., Mathews F.S., Sly W.S., Grubb J.H. (1996). Structure of human β-glucuronidase reveals candidate lysosomal targeting and active-site motifs. Nat. Struct. Biol..

[5] Wallace B.D., Wang H., Lane K.T., Scott J.E., Orans J., Koo J.S., Venkatesh M., Jobin C., Yeh L.-A., Mani S. (2010). Alleviating cancer drug toxicity by inhibiting a bacterial enzyme. Science.

[6] Pusztaszeri M.P., Genta R.M., Cryer B.L. (2007). Drug-induced injury in the gastrointestinal tract: Clinical and pathologic considerations. Nat. Clin. Pract. Gastroenterol. Hepatol..

[7] Zhou X., Wang D., Gao Z., He M., Hou J., Zhang H., Zhang G., Ding D., Feng G. (2022). Highly sensitive light-up near-infrared fluorescent probe for detection and imaging of β-glucuronidase in human serum, living cells and tumor-bearing mice. Sci. China Mater..

[8] Houba P.H.J., Boven E., van der Meulen-Muileman I.H., Leenders R.G.G., Scheeren J.W., Pinedo H.M., Haisma H.J. (2001). Pronounced antitumor efficacy of doxorubicin when given as the prodrug DOX-GA3 in combination with a monoclonal antibody β-glucuronidase conjugate. Int. J. Cancer.

[9] Cao T.Q., Phong N.V., Kim J.H., Gao D., Anh H.L., Ngo V.-D., Vinh L.B., Koh Y.S., Yang S.Y. (2021). Inhibitory effects of cucurbitane-type triterpenoids from *Momordica charantia* fruit on lipopolysaccharide-stimulated pro-inflammatory cytokine production in bone marrow-derived dendritic cells. Molecules.

[10] Van Cong P., Anh H.L.T., Trung N.Q., Quang Minh B., Viet Duc N., Van Dan N., Trang N.M., Phong N.V., Vinh L.B., Anh L.T. (2022). Isolation, structural elucidation and molecular docking studies against SARS-CoV-2 main protease of new stigmastane-type steroidal glucosides isolated from the whole plants of *Vernonia gratiosa*. Nat. Prod. Res..

[11] Hwang Y.-H., Ha H., Ma J.Y. (2013). Acute oral toxicity and genotoxicity of *Dryopteris crassirhizoma*. J. Ethnopharmacol..

[12] Shinozaki J., Shibuya M., Masuda K., Ebizuka Y. (2008). Dammaradiene synthase, a squalene cyclase, from *Dryopteris crassirhizoma* Nakai. Phytochemistry.

[13] Yang Q., Gao L., Si J., Sun Y., Liu J., Cao L., Feng W.-H. (2013). Inhibition of porcine reproductive and respiratory syndrome virus replication by flavaspidic acid AB. Antivir. Res..

[14] Lee S.-M., Na M.-K., An R.-B., Min B.-S., Lee H.-K. (2003). Antioxidant activity of two phloroglucinol derivatives from *Dryopteris crassirhizoma*. Biol. Pharm. Bull..

[15] Lee H.B., Kim J.C., Lee S.M. (2009). Antibacterial activity of two phloroglucinols, flavaspidic acids AB and PB, from *Dryopteris crassirhizoma*. Arch. Pharm. Res..

[16] Lee J.S., Miyashiro H., Nakamura N., Hattori M. (2008). Two new triterpenes from the rhizome of *Dryopteris crassirhizoma*, and inhibitory activities of its constituents on human immunodeficiency virus-1 protease. Chem. Pharm. Bull..

[17] Chang S.-H., Bae J.-H., Hong D.-P., Choi K.-D., Kim S.-C., Her E., Kim S.-H., Kang C.-D. (2010). *Dryopteris crassirhizoma* has anti-cancer effects through both extrinsic and intrinsic apoptotic pathways and G0/G1 phase arrest in human prostate cancer cells. J. Ethnopharmacol..

[18] Yim N.-H., Lee J.-J., Lee B., Li W., Ma J.Y. (2019). Antiplatelet activity of acylphloroglucinol derivatives isolated from *Dryopteris crassirhizoma*. Molecules.

[19] Han X., Li Z., Li C.-Y., Jia W.-N., Wang H.-T., Wang C.-H. (2015). Phytochemical constituents and biological activities of plants from the genus *Dryopteris*. Chem. Biodivers..

[20] Yuk H.J., Kim J.-Y., Sung Y.-Y., Kim D.-S. (2021). Phloroglucinol derivatives from *Dryopteris crassirhizoma* as potent xanthine oxidase inhibitors. Molecules.

[21] Pham V.C., Kim O., Lee J.-H., Min B.S., Kim J.A. (2017). Inhibitory effects of phloroglucinols from the roots of *Dryopteris crassirhizoma* on melanogenesis. Phytochem. Lett..

[22] Wang J., Yan Y.-T., Fu S.-Z., Peng B., Bao L.-L., Zhang Y.-L., Hu J.-H., Zeng Z.-P., Geng D.-H., Gao Z.-P. (2017). Anti-influenza virus (H5N1) activity screening on the phloroglucinols from rhizomes of *Dryopteris crassirhizoma*. Molecules.

[23] Phong N.V., Oanh V.T., Yang S.Y., Choi J.S., Min B.S., Kim J.A. (2021). PTP1B inhibition studies of biological active phloroglucinols from the rhizomes of *Dryopteris crassirhizoma*: Kinetic properties and molecular docking simulation. Int. J. Biol. Macromol..

[24] Lineweaver H., Burk D. (1934). The determination of enzyme dissociation constants. J. Am. Chem. Soc..

[25] Dixon M. (1953). The determination of enzyme inhibitor constants. Biochem. J..

[26] Taha M., Ismail N.H., Imran S., Selvaraj M., Rashwan H., Farhanah F.U., Rahim F., Kesavanarayanan K.S., Ali M. (2015). Synthesis of benzimidazole derivatives as potent *β*-glucuronidase inhibitors. Bioorg. Chem..

[27] Roberts A.B., Wallace B.D., Venkatesh M.K., Mani S., Redinbo M.R. (2013). Molecular insights into microbial *β*-glucuronidase inhibition to abrogate CPT-11 toxicity. Mol. Pharmacol..

[28] Kuntz I.D., Blaney J.M., Oatley S.J., Langridge R., Ferrin T.E. (1982). A geometric approach to macromolecule-ligand interactions. J. Mol. Biol..

[29] Viet Phong N., Thi Nguyet Anh D., Yeong Chae H., Young Yang S., Jeong Kwon M., Sun Min B., Ah Kim J. (2022). Anti-inflammatory activity and cytotoxicity against ovarian cancer cell lines by amide alkaloids and piperic esters isolated from *Piper longum* fruits: In vitro assessments and molecular docking simulation. Bioorg. Chem..

[30] Torres P.H.M., Sodero A.C.R., Jofily P., Silva-Jr F.P. (2019). Key topics in molecular docking for drug design. Int. J. Mol. Sci..

[31] Kim J.H., Vinh L.B., Hur M., Koo S.-C., Park W.T., Moon Y.-H., Lee Y.J., Kim Y.H., Huh Y.-C., Yang S.Y. (2021). Inhibitory activity of 4-*O*-benzoyl-3′-*O-*(*O*-methylsinapoyl) sucrose from *Polygala tenuifolia* on *Escherichia coli β*-glucuronidase. J. Microbiol. Biotechnol..

[32] Morris G.M., Huey R., Lindstrom W., Sanner M.F., Belew R.K., Goodsell D.S., Olson A.J. (2009). AutoDock4 and AutoDockTools4: Automated docking with selective receptor flexibility. J. Comput. Chem..

[33] Lin H.-Y., Chen C.-Y., Lin T.-C., Yeh L.-F., Hsieh W.-C., Gao S., Burnouf P.-A., Chen B.-M., Hsieh T.-J., Dashnyam P. (2021). Entropy-driven binding of gut bacterial β-glucuronidase inhibitors ameliorates irinotecan-induced toxicity. Commun. Biol..

